# Radioprotective potential of whey protein against gamma irradiation-induced lingual damage

**DOI:** 10.3389/fphar.2023.1293230

**Published:** 2023-12-13

**Authors:** Hanaa M. Hassan, Asmaa M. Abdeen, Ibrahim Y. Abdelrahman, Walied Abdo, Saher S. Mohammed, Ahmed Abdeen, Afaf Abdelkader, Rada Olga, Liana Fericean, Samah F. Ibrahim, Heba I. Ghamry, Farouk S. Elgendy, Safwa M. Sorour, Abeer A. Eldeeb, Osama Ahmed, Fatema Rashed, Maha M. Bikheet

**Affiliations:** ^1^ Department of Agricultural Chemistry, Faculty of Agriculture, Minia University, Minia, Egypt; ^2^ Department of Oral Biology, Faculty of Dentistry, Minia University, Minia, Egypt; ^3^ Department of Radiation Biology, National Centre for Radiation Research and Technology (NCRRT), Egyptian Atomic Energy Authority (EAEA), Cairo, Egypt; ^4^ Department of Pathology, Faculty of Veterinary Medicine, Kafrelsheikh University, Kafrelsheikh, Egypt; ^5^ Department of Forensic Medicine and Toxicology, Faculty of Veterinary Medicine, Benha University, Toukh, Egypt; ^6^ Department of Forensic Medicine and Clinical Toxicology, Faculty of Medicine, Benha University, Benha, Egypt; ^7^ Department of Biology and Plant Protection, Faculty of Agriculture, University of Life Sciences, Timișoara, Romania; ^8^ Department of Clinical Sciences, College of Medicine, Princess Nourah bint Abdulrahman University, Riyadh, Saudi Arabia; ^9^ Nutrition and Food Sciences, Department of Home Economics, Faculty of Home Economics, King Khalid University, Abha, Saudi Arabia; ^10^ Department of Pharmacology, Faculty of Medicine, Benha University, Benha, Egypt; ^11^ Department of Anatomy and Embryology, Faculty of Veterinary Medicine, Benha University, Toukh, Egypt; ^12^ Department of Basic Medical and Dental Science, Faculty of Dentistry, Zarqaa University, Zarqaa, Jordan; ^13^ Dairy Science Department, Faculty of Agriculture, Minia University, Minia, Egypt

**Keywords:** ionizing radiation, whey protein, inflammation, mRNA expression, oxidative damage, tongue

## Abstract

**Introduction:** Ionizing radiation (IR) is effectively used in the treatment of oral malignancies; however, it might also significantly harm the surrounding tissues. Whey protein isolate (WP) is a protein derived from milk that exhibits a wide range of bioactivities. Therefore, the present research aimed to delineate the mitigating impact of WP against gamma irradiation-induced lingual damage.

**Methods:** Rats were randomized into 5 groups: Control (saline, orally, 14 days), WP (WP; 0.5 g/kg b. w., orally, 14 days), IR (saline, orally, 14 days, exposed to 6 and 3 Gy on days 4 and 6, respectively), WP+IR (WP was given orally for 14 days before and after IR exposure; exposed to 6 and 3 Gy on days 4 and 6, respectively), and IR+WP (WP, orally, started 24 h after 1^st^ IR exposure till the end of the experiment) groups. Samples were collected at two-time intervals (on the 7^th^ and 14^th^ days).

**Results and Discussion:** Oxidative stress was stimulated upon IR exposure in tongue, indicated by boosted malondialdehyde (MDA) level, along with a decrease in the total antioxidant capacity (TAC) level, superoxide dismutase (SOD), and catalase (CAT) activities. Additionally, IR exposure depicted an increase of serum IgE, inflammatory cytokines, including tumor necrosis factor-α (TNF-α), interleukin (IL)-6, along with overexpression mRNA levels of nuclear factor kappa-B transcription factor/p65 (NF-κB/p65), and down-regulation of nuclear factor erythroid 2–related factor 2 (NRF2) and heme oxygenase (HO-1) mRNA levels in tongue tissue. Moreover, IR triggered alterations in lingual histological architecture. The antioxidant and anti-inflammatory properties of WP mitigated oxidative damage, inflammation, and desquamation that were brought on following IR exposure. The protective administration of WP markedly decreases IR-induced lingual harm compared to the mitigation protocol. Our findings recommend WP supplements to the diets of cancer patients undergoing IR that might aid radioprotective effects.

## 1 Introduction

Radiotherapy is the utilization of ionizing radiation (IR) to treat some benign, primary, or metastatic malignant tumors of the central nervous system and autoimmune disorders, such as thyroid eye disease (De Ruysscher et al., 2019). Currently, IR is an important part of several head and neck tumor treatments. This region is an intricate area made up of various distinctive structures, including the skin, mucosa, salivary gland, teeth, bone, and cartilage that respond to radiation differently, especially in patients who are exposed to higher doses of IR over large areas (Pandya et al., 2014). It has been associated with several oral adverse consequences, such as xerostomia, mucositis, taste alteration, and mucosal ulceration (Deshpande et al., 2018).

IR can directly hit DNA or indirectly by increasing the generation of free radicals, i.e., oxidative stress (Leroi et al., 2016; De Ruysscher et al., 2019; Allegra et al., 2020). The cytotoxic effects of IR on normal cells are essentially due to the radiolysis of cellular water, leading to instant ionization of water molecules, producing free radicals including reactive oxygen species (ROS) and nitrogen species (RNS), such as hydrogen peroxide (H_2_O_2_), superoxide anion (O_2_^•-^), hydroxyl radical (OH^•^), and nitric oxide (NO). If not scavenged, this ROS/RNS causes cell injury by triggering mitochondrial perturbation, lipid peroxidation (LPO), DNA strand breakage, protein misfolding, and, eventually, apoptosis (Allegra et al., 2020). Furthermore, since oxidative stress and inflammation are strongly related, inflammation is one of the most significant mechanisms that follow IR exposure (Citrin and Mitchell, 2017). It is anticipated that ROS and DNA damage will initiate an intracellular signaling cascade and enhance the release of inflammatory mediators (Shanab et al., 2023). Therefore, the main approach to radiation mitigation is the scavenging of free radicals and modulation of the inflammatory response. The exploration of novel natural radioprotective compounds based on these mechanisms to withstand IR-induced tissue damage and promote tissue renewal is of utmost interest.

Whey-derived dairy proteins have recently garnered great attention not only for their nutritional benefits but also for their biological capabilities (Corrochano et al., 2018). Whey protein (WP) isolates are proteins derived from milk following enzymatic treatment or the addition of organic acids or minerals with the precipitation and removal of casein (Costa et al., 2021). They serve as an excellent source of proteins, such as lactoglobulin, peptone protease, immunoglobulins, albumin, lactalbumin, lactoferrin, lactoperoxidase, bovine serum, and peptides (Corrochano et al., 2018; Giblin et al., 2019). These proteins contain a variety of sulfur-containing amino acids, including valine, isoleucine, and leucine, and branched-chain amino acids, such as cysteine and methionine, which are necessary for tissue growth and repair (Sukkar and Bounous, 2004). WPs are known as “fast proteins” because they do not clot in the stomach’s acidic environment and can be easily digested and absorbed, especially for cancer IR patients with poor digestive function (Giblin et al., 2019). WP exhibits a wide range of biological activities, such as antioxidant, anti-inflammatory, antihypertensive (Pal and Ellis, 2010), and immunomodulatory properties (Giblin et al., 2019). The antioxidant potential of WP is attributed to its role as a precursor for GSH, the body’s main antioxidant against ROS (Ali et al., 2022). Furthermore, WP exerts an anti-inflammatory effect by inhibiting the pro-inflammatory cytokines and mediators, which may be regulated by the NF-κB signaling pathways (Ali et al., 2022).

In line with this affirmation, it is a worthy point of investigation to assess the radiotoxic effect on the rat tongue and study the possible radioprotective and mitigating effects of WP. Therefore, a sub-lethal dose of irradiation was employed, and evaluation of the serum cytokines, lingual oxidative stress parameters, histomorphology, and mRNA expression of oxidative and pro-inflammatory factors was performed in our study.

## 2 Materials and methods

### 2.1 Whey protein isolate preparation and Fourier-transform infrared spectroscopy

Cow whey protein powder was obtained from the Department of Dairy Sciences, Faculty of Agriculture, Minia University, Egypt. WP isolate was prepared as described in Jahovic et al. (2005). All spectra were obtained using Fourier-transform infrared (FTIR) spectroscopy (Singh et al., 2011).

### 2.2 Irradiation source and technique

The source of radiation was a Gammacell 40 (cesium-137) installed in the National Center for Research and Radiation Technology (NCRRT), Nasr City, Cairo, Egypt, which ensures a homogeneous distribution of whole-body irradiation. Rats were housed in cages in the gamma irradiation chamber. Rats were exposed to 6 and 3 Gy at a dosage rate of 3 Gy/min.

### 2.3 Animals and design of the trial

Forty adult male Sprague–Dawley rats (weighing 160–180 g) were employed in this study. They were obtained from the animal house of the Atomic Energy Authority, Cairo, Egypt. The rats were housed, allowed to acclimatize to the ambient conditions (25°C ± 2°C), and granted free access to water and a balanced pelleted ratio of 25.8% protein, 62.8% carbohydrates, and 11.4% fat (Wadi Co., Giza, Egypt).

**FIGURE 1:**
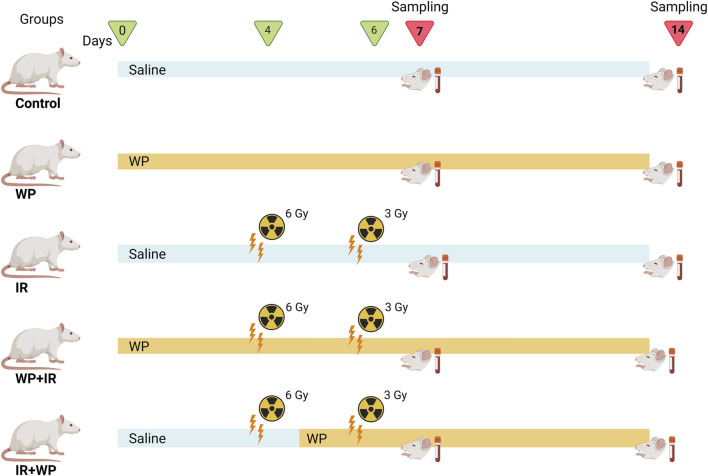
Experimental protocol.

As illustrated in Figure 1, rats were randomized into five groups, comprising eight rats in each group: 1) in the control group, rats were given saline only by oral gavage, daily throughout the experimental period. 2) In the WP group, rats received freshly prepared WP daily at a dose of 0.5 g/kg b.w., orally (Jahovic et al., 2005) for 14 days (days 0–13). 3) In the IR group, animals were exposed to two doses of whole-body radiation; the first irradiation of 6 Gy on day 4 and the second irradiation of 3 Gy on day 6, with a total of 9 Gy accumulative dose, which was determined according to El-Rouby and El-Batouti, (2021) with some modifications. 4) In the WP + IR group, rats received a daily dose of WP (0.5 g/kg b.w., orally) for 14 days (days 0–13), prior and post-irradiation IR exposures. 5) In the IR + WP group, rats received WP (0.5 g/kg/day b.w., orally) 24 h after the first IR exposure until the end of the experiment.

### 2.4 Specimen collection and processing

At the completion of the experiment, the rats were humanely euthanized by giving thiopental sodium (50 mg/kg) intraperitoneally (Abdelrahman et al., 2020). At 24 h after dosing of saline or WP, the samples were collected at two time intervals (on days 7 and 14). Blood samples were drawn from the hepatic vein and centrifuged for 15 min at 3,000 × g, and the sera were gathered and preserved at −20°C for further IgE and cytokine analyses. Tongue specimens from each rat were swiftly dissected and washed out using ice-cold physiological saline to get rid of any clogs and, thereafter, divided into numerous portions. For histological examination, one portion was preserved in 10% neutral buffered formalin (El-Nasr Company for Intermediate Chemicals, Giza, Egypt). Other portions of tissue were stored at −80°C and further used for estimation of oxidative biomarkers and RNA extraction.

### 2.5 Biochemical index bioassay

ELISA kits were used for determining the serum levels of histamine (Cat. No.: K4163, BioVision, Inc.), immunoglobulin E (IgE, Cat. No.: CSB-E07984r, CUSABIO Technology LLC, TX, United States), tumor necrosis factor-alpha (TNF-α, Cat. No.: MBS175904, MyBioSource, Inc., CA, United States), and interleukin 6 (IL-6, Cat. No.: R6000B, R&D System, MN, United States). All procedures were performed as described by the manufacturers.

### 2.6 Evaluation of antioxidants and peroxidation biomarker

Malondialdehyde (MDA; an LPO marker) and the total antioxidant capacity (TAC) concentrations, along with the antioxidant enzyme activities (catalase (CAT) and superoxide dismutase (SOD)), were assessed in the lingual tissue homogenate, following the manufacturer’s guide (Laboratory Biodiagnostics, Cairo, Egypt).

### 2.7 Quantitative real-time PCR

Quantitative real-time PCR (qRT-PCR) was conducted to determine the mRNA expression of nuclear factor-κB (NF-κB/p65), erythroid 2-related factor 2 (NRF2), and heme oxygenase-1 (HO-1). In brief, RNA was isolated from the tongue tissue using TRIzol reagent, following the manufacturer’s instructions of the RNA Purification Kit (Thermo Fisher Scientific, United States). A NanoDrop spectrophotometer (Thermo Fisher Scientific, United States) was used to measure RNA. Then, RNA extracts with A260/A280 ≥ 1.8 were reverse-transcribed into cDNA employing a High-Capacity cDNA Reverse Transcription Kit (Thermo Fisher Scientific, United States). The PCR cycle procedures were conducted as described in Habotta et al. (2023). The primer sequences and gene accession codes for the evaluation of NRF2, HO-1, and NF-κB/p65 gene expression levels are listed in Table 1. The gained data were assessed utilizing the 2^−ΔΔCT^ method.

**TABLE 1:** Primers used for qRT-PCR.

Gene	Forward sequence (5′-3′)	Reverse sequence (5′-3′)	Accession No.
NRF2	TTG​TAG​ATG​ACC​ATG​AGT​CGC	TGT​CCT​GCT​GTA​TGC​TGC​TT	NM_031789.2
HO-1	GTA​AAT​GCA​GTG​TTG​GCC​CC	ATG​TGC​CAG​GCA​TCT​CCT​TC	NM_012580.2
NF-κB/p65	TTC​CCT​GAA​GTG​GAG​CTA​GGA	CAT​GTC​GAG​GAA​GAC​ACT​GGA	NM_199,267.2
β-Actin	AGG​AGT​ACG​ATG​AGT​CCG​GC	CGC​AGC​TCA​GTA​ACA​GTC​CG	NM 031144

### 2.8 Histoarchitecture inspection

The samples of lingual tissue fixed with formalin were first dehydrated in escalating alcohol concentration. Afterward, xylene clearance was performed before embedding in paraffin. To examine the histoarchitecture, the tissue was sliced into 5-µm-thick sections, then stained with hematoxylin and eosin (H&E), and imaged using a camera-integrated digital imaging system (DM300, Leica, Germany). All reagents used for dehydration, paraffin-embedding, and staining were obtained from Laboratory Biodiagnostics, Cairo, Egypt.

Next, the slides were examined blindly for assessment of the oral damage. Each examined slide was scored and graded upon the following criteria: grade 0 showed normal mucosa with normal papillae, normal submucosal glands, and normal striated muscle; grade I showed mild degeneration of the filiform papillae with intact root, mild hyperkeratosis, atrophy of taste buds (up to 20% loss of taste cells), fungiform, circumvallate papillae, submucosal edema, and mild sarcoplasmic degeneration of the striated muscle; grade II showed sloughing roots of filiform papillae, moderate increase in the previous criteria with 21%–40% loss of the taste cells, edema extended deep to the muscle layer which demonstrated marked atrophy, separation of the muscle fibers, and infiltration of mast cells; grade III showed complete sloughing of the filiform papillae with marked hyperkeratosis, 21%–60% loss of the taste cells, associated fragmentation of the muscle fibers, and an increase in mast cell infiltration; grade IV showed marked development of the degenerative and desquamation of the oral filiform papillae and keratin layer, more than 60% loss of taste cells, severe edema extended between the degenerated and fragmented muscle fibers, and marked infiltration of mast cells.

### 2.9 Statistical analysis

The data were analyzed using one-way analysis of variance (ANOVA), and Duncan’s *post hoc* test was used to compare the means of the treatment groups. First, all data were tested for normality (Shapiro–Wilk’s test; *p* > 0.05) and homogeneity of variances (Levene’s test; *p* > 0.05) before conducting the ANOVA. All values are deemed statistically significant at *p* < 0.05 and stipulated as the mean and 95% confidence interval. Univariate data visualization was done using OriginPro software (version 2019b, OriginLab, MA, United States). The MetaboAnalyst program was also used to create biological networks, hierarchical heatmap, and variable importance in projection (VIP) score.

## 3 Results

### 3.1 FTIR

**FIGURE 2:**
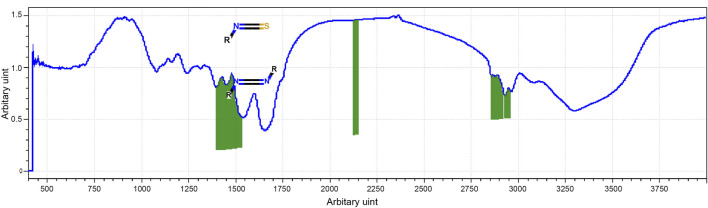
FTIR spectra of WP.

The most prevalent secondary structural components of protein are α-helix and β-sheet, which have been identified using FTIR spectroscopy of the whey protein. The amide-I region of FTIR spectra showed stretching vibrations of C=O and C–N groups and were directly related to protein secondary structures while the region represented C–H bending. The amide-I band (2,130–2,160 cm^−1^) was generated (Figure 2).

### 3.2 Effect of WP on the oxidative status in IR-exposed tongue tissue

**FIGURE 3:**
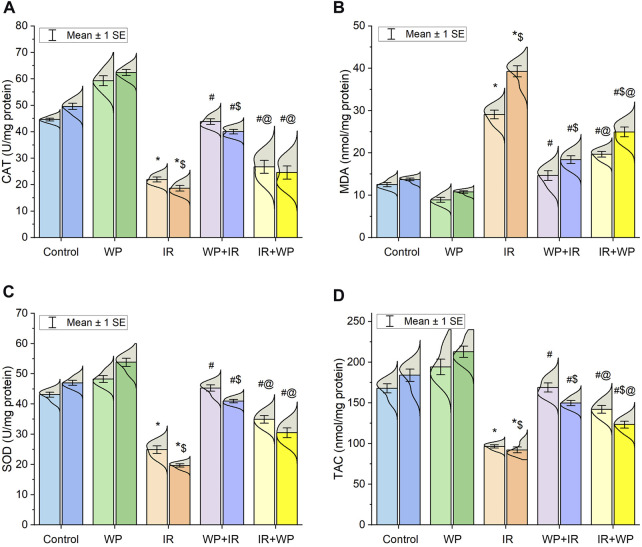
Bar plot panel of oxidant/antioxidant indices upon IR exposure and/or WP supplementation in lingual tissues. **(A)** Catalase enzyme, CAT; **(B)** malondialdehyde, MDA; **(C)** superoxide dismutase enzyme, SOD; and **(D)** total antioxidant capacity, TAC. Light- and dark-colored columns represent days 7 and 14, respectively, within the same group. Values are presented as mean ± SE (n = 4 for each time point per group; *p* < 0.05). * IR vs. control group. # WP+IR or IR+WP vs. IR group. $ day 7 vs. day 14 means within the same group. @ WP+IR vs. IR+WP group.

IR-induced oxidative stress in the rat tongue was indicated, as evidenced by a decrease in the CAT and SOD activities and TAC content, along with an increase in MDA levels. Interestingly, it was demonstrated that the degree of IR-induced oxidative damage displayed a time-dependent pattern, and it increased on day 14 compared to day 7. In contrast, the supplementation of WP improved the antioxidant status after days 7 and 14, as shown in Figure 3. We noticed an improvement in oxidative stress and LPO in WP + IR compared to the IR + WP and IR groups.

**FIGURE 4:**
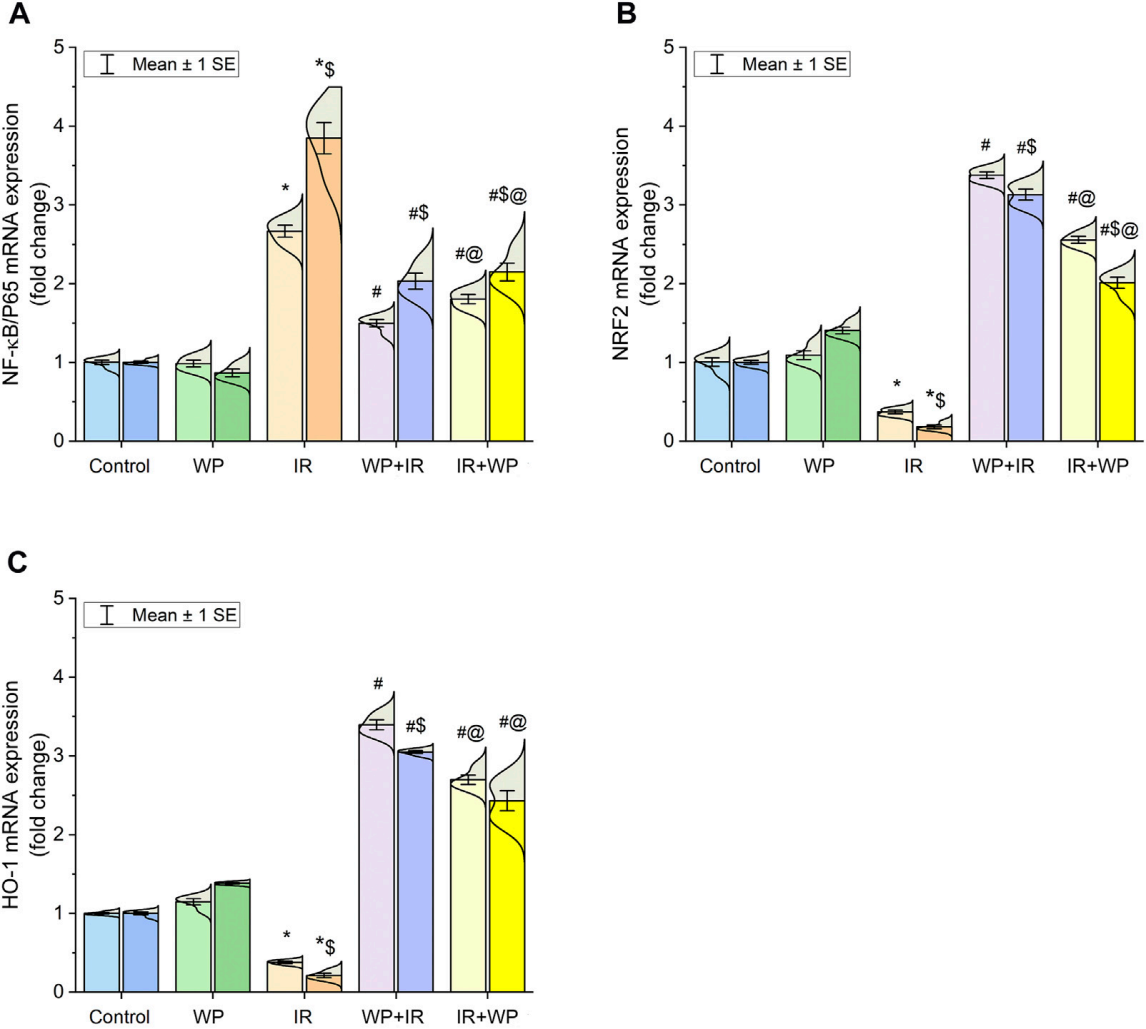
Bar plot panel of mRNA expression of antioxidant and inflammation-related genes following IR exposure and/or WP supplementation in lingual tissues. **(A)** Nuclear factor kappa-B transcription factor/p65, NF-κB/p65; **(B)** nuclear factor erythroid 2-related factor 2, NRF2; **(C)** heme oxygenase-1, HO-1. Light- and dark-colored columns represent days 7 and 14, respectively, within the same group. Values are presented as mean ± SE (n = 4 for each time point per group; *p* < 0.05). * IR vs. control group. # WP+IR or IR+WP vs. IR group. $ day 7 vs. day 14 means within the same group. @ WP+IR vs. IR+WP group.

In addition, the lingual gene expression levels of the NRF2 and HO-1 in exposed rats were analyzed. Compared to the control, the irradiated group exhibited downregulation in the mRNA expression of both antioxidant mediators (Figure 4). In contrast, WP administration aroused upregulation in their gene expression levels in relation to IR exposure. Pretreatment with WP elicited an optimum improvement in the NRF2 and HO-1 expression levels compared to post-exposure treatment with upsurge in their expression at day 14 after irradiation compared to day 7 in a time-dependent manner.

### 3.3 Effect of WP on the inflammation of the IR-exposed tongue

**FIGURE 5:**
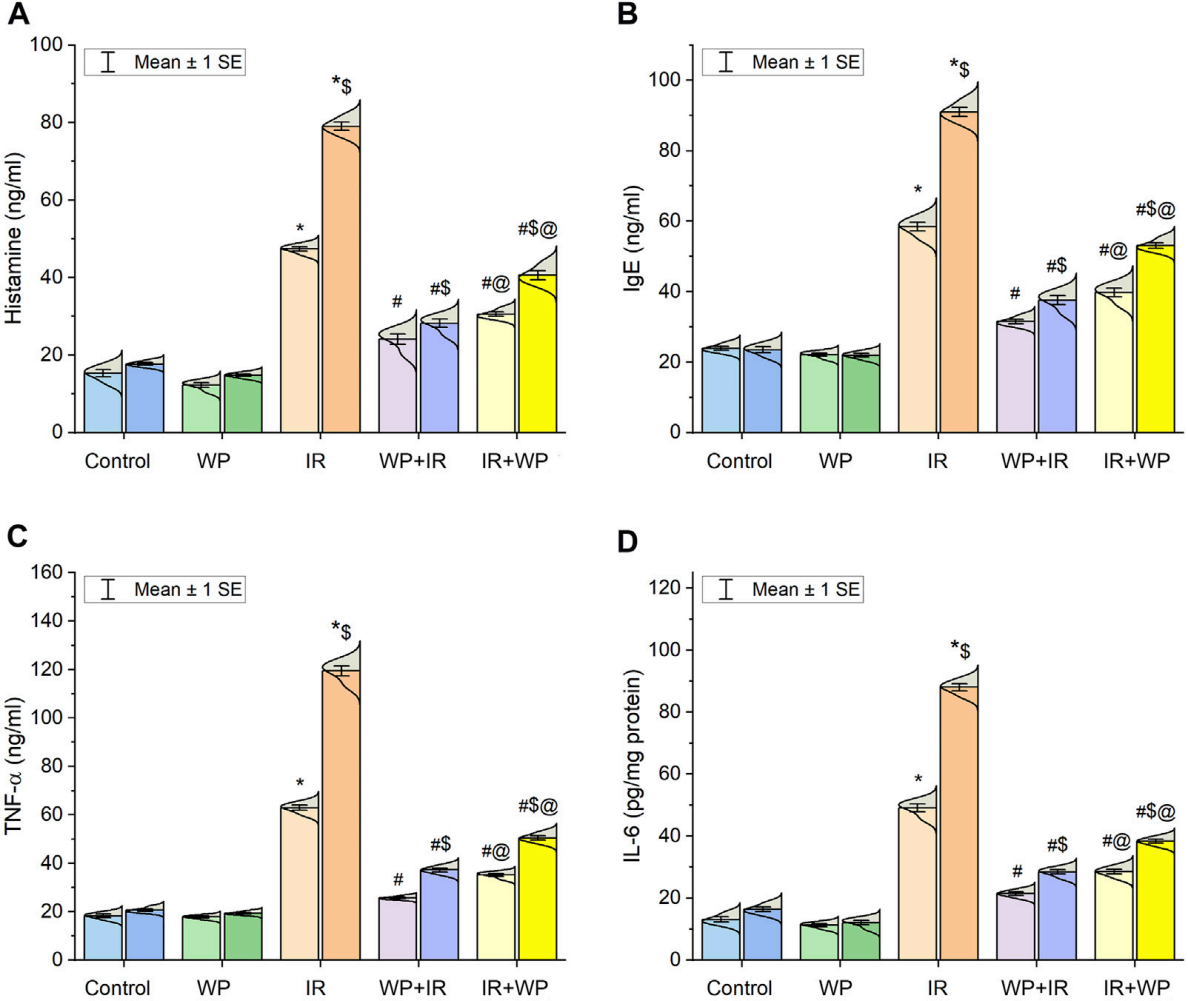
Bar plot panel of serum levels of inflammatory markers following IR exposure and/or WP supplementation. **(A)** Histamine; **(B)** immunoglobulin E, IgE; **(C)** tumor necrosis factor-α, TNF-α; and **(D)** interleukin-6, IL-6. Light- and dark-colored columns represent days 7 and 14, respectively, within the same group. Values are presented as mean ± SE (n = 4 for each time point per group; *p* < 0.05). * IR vs. control group. # WP+IR or IR+WP vs. IR group. $ day 7 vs. day 14 means within the same group. @ WP+IR vs. IR+WP group.

As also shown in Figure 4, significant upregulation of the mRNA expression of NF-κB/p65 was noticed in the lingual mucosa of the IR-exposed group. Additionally, as shown in Figure 5, IR exposure evoked a systemic injury, proven by an increase in serum levels of histamines, IgE, and cytokines, involving TNF-α and IL-6, in relation to the control and WP groups. These levels significantly increased at day 14 compared to day 7 post-irradiation in a time-dependent pattern. However, the results were reversed in groups that received WP, and its administration before IR exposure was capable of eliciting a stronger anti-inflammatory impact than that of post-IR treatment. According to these findings, WP might be able to alleviate inflammation including that occurred in the lingual tissue following IR toxicity.

### 3.4 Histoarchitecture findings

**FIGURE 6:**
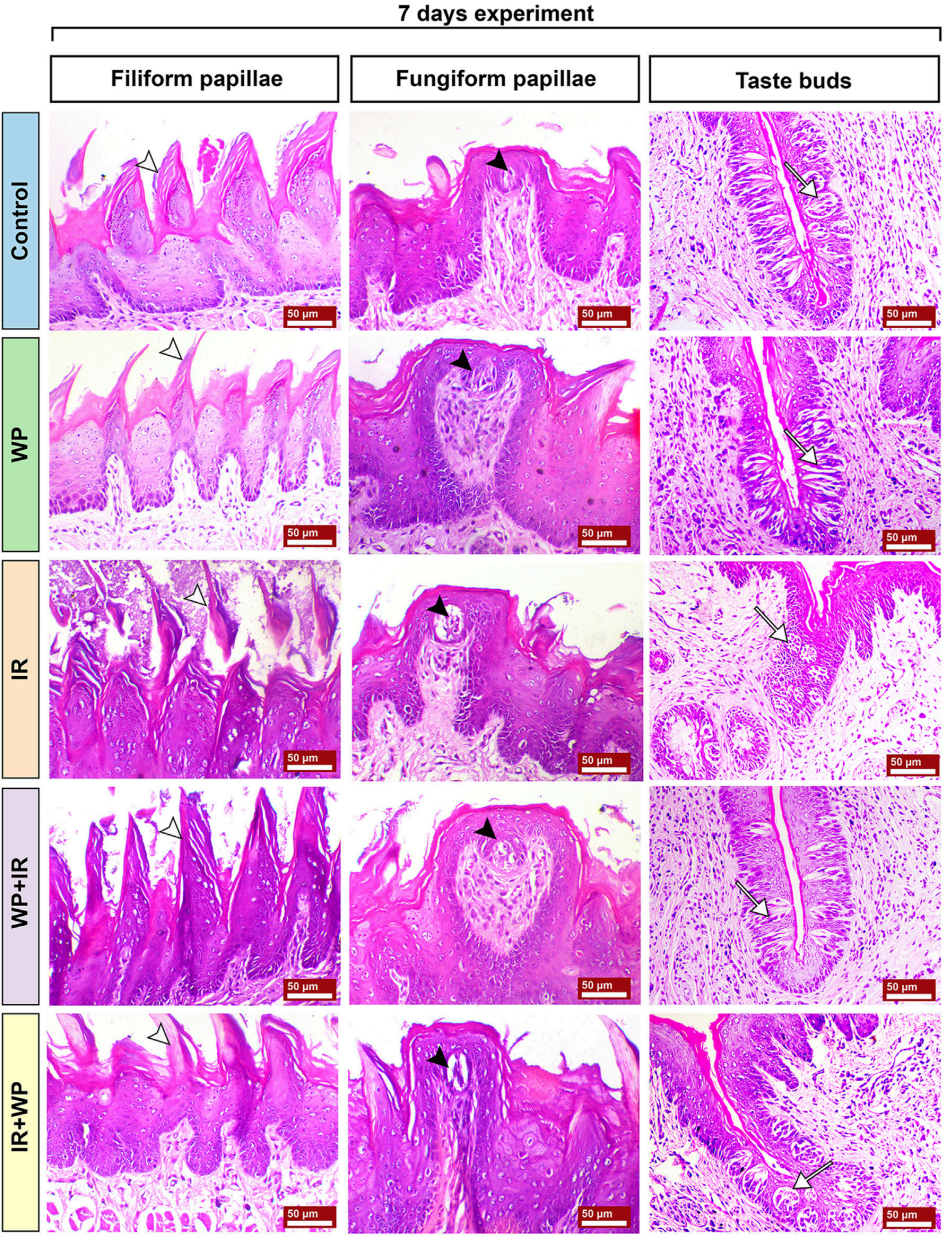
Photomicrograph of tongue papillae from different treatment groups of rats sacrificed at 7 days post-irradiation. Control and WP groups show a gross normal architecture. Both show normal filiform papillae (white arrowhead), normal fungiform papillae with intra-epithelial taste buds on top of the papillae (black arrowhead), and morphologically normally arranged intra-epithelial taste buds on both sides of circumvallate papillae (white arrow). The IR group exhibits severe keratinolysis associated with sloughing of the filiform papillae (white arrowhead), keratinolysis, loss of the cells of taste buds of fungiform papillae (black arrowhead), and necrosis of the taste buds in circumvallate papillae (white arrow). The WP+IR group reveals that filiform papillae retained its eosinophilic keratin layer and basophilic keratohyalin granules (white arrowhead), less atrophied fungiform papillae with intra-epithelial taste buds on top of the papillae (black arrowhead), and few apoptosis of taste bud cells on both sides of the circumvallate papillae (white arrow). The IR+WP group shows filiform papillae with conical shape (white arrowhead) and fungiform papillae with atrophied taste buds (black arrowhead), and there is a decrease in the atrophy of the taste buds of the circumvallate papillae (white arrow). H&E stain, bars = 50 µm.

**FIGURE 7:**
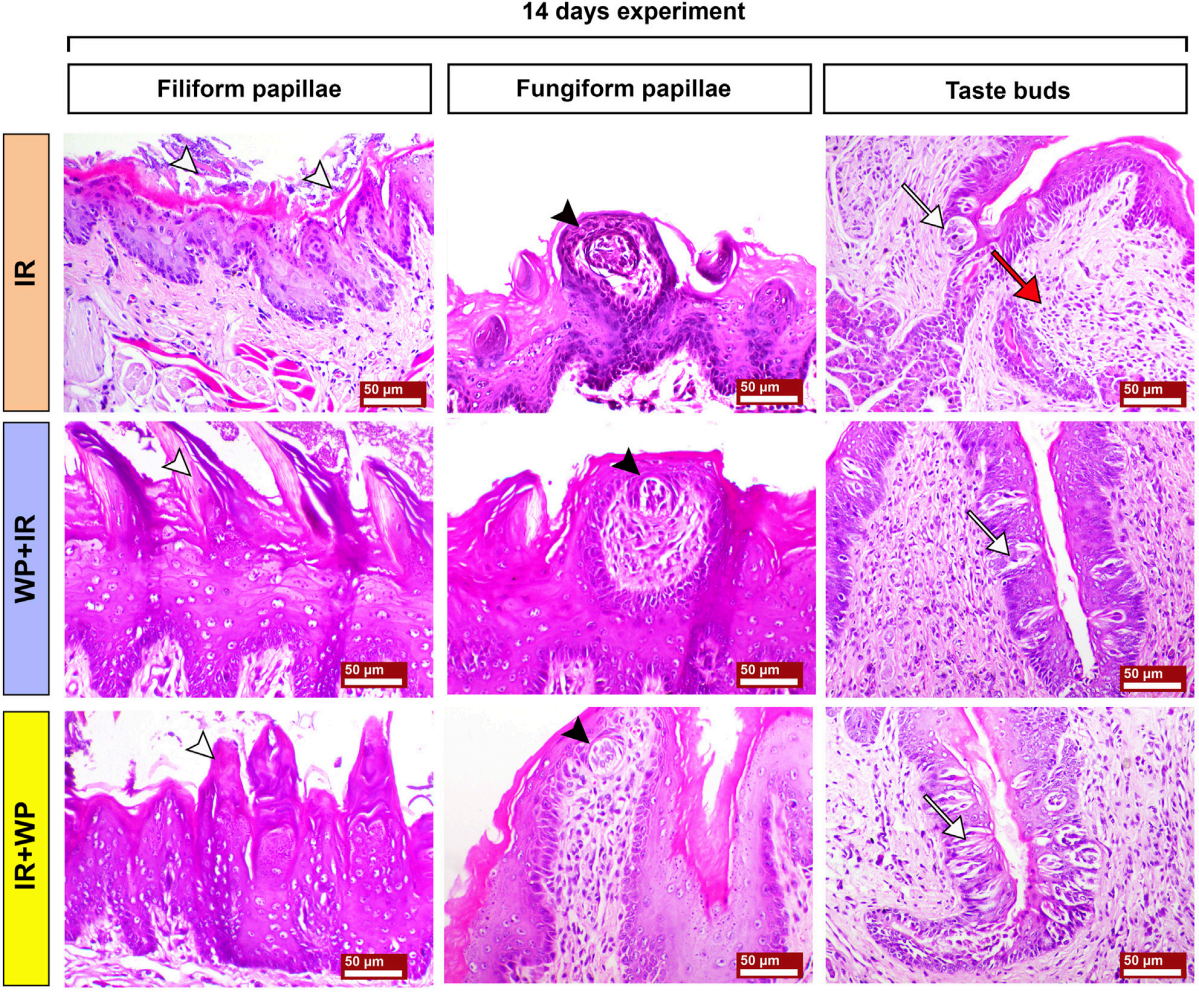
Photomicrographs of tongue papillae from different treatment groups of rats sacrificed at 14 days post-irradiation. The IR group shows the complete loss of the filiform papillae (white arrowhead) and atrophy of the fungiform papillae with disorganized taste bud cells (black arrowhead). The circumvallate papillae show severe atrophy of their taste buds (white arrow), stagnated excretory duct of the von Ebner salivary gland, and periductal mononuclear inflammatory cell infiltration (red arrow). The WP+IR group displays a retained basophilic keratin layer and the conical shape of the filiform papillae (white arrowhead), fungiform papillae with less atrophied taste buds (black arrowhead), and apoptotic cells of taste buds (white arrow). The IR+WP group demonstrates few cone-shaped filiform papillae (white arrowhead), fungiform papillae losing its normal shape with mild atrophy of the taste buds (black arrowhead), and less atrophic changes in the taste buds of the circumvallate papillae (white arrow). H&E stain, bars = 50 µm.

**FIGURE 8:**
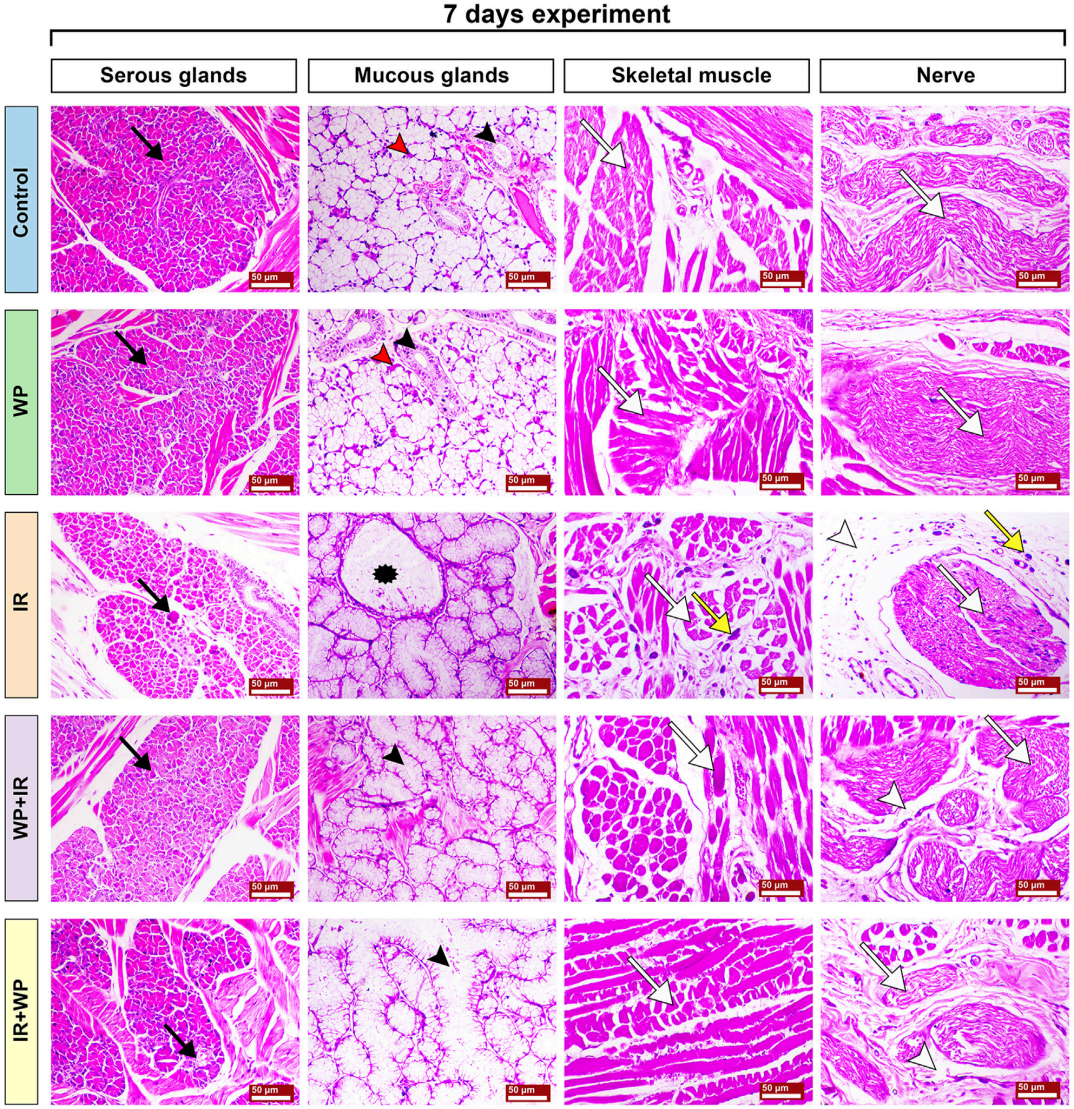
Photomicrographs of the subepithelial structures of the tongues of different groups of rats sacrificed at 7 days post-irradiation. Control and WP groups show a normal sublingual tissue architecture. The serous acini reveal normal pyramidal cells with basally located nuclei and intercalated ducts (black arrow). The mucous acini show flat basally located nuclei, intralobular striated ducts (red arrowhead), and serous demilunes (red arrowhead). The striated muscles of the tongue show normal bundles of muscle fibers (white arrow). Zigzag-shaped nerve fibers form nerve bundles surrounded by the epineurium and separated by connective tissue (white arrow). The IR group exhibits severe degeneration and apoptosis of the serous acini (black arrow), dilatation of the glands with excess mucous (asterisk), severe atrophied and degenerated muscle fibers (white arrow) associated with marked infiltration of mast cells (yellow arrow), atrophied nerve bundles and fibers (white arrow) accompanied with marked infiltration of mast cells (yellow arrow), and edema (white arrowhead). The WP+IR group shows mild apoptosis of the serous glands (black arrow), slightly dilated mucous acini (black arrowhead), a decrease in necrotic changes within the muscle fibers (white arrow), and normal nerve bundles (white arrow) with a remarkable decrease in edema (white arrowhead). The IR+WP group displays focal eosinophilic degenerative changes within the serous glands (black arrow), moderately dilated mucous acini (black arrowhead), fragmentation of the muscle fibers (white arrow), and slight signs of separation and degeneration of the nerve bundles (white arrow) with a decrease in edema (white arrowhead). H&E stain, bars = 50 µm.

**FIGURE 9:**
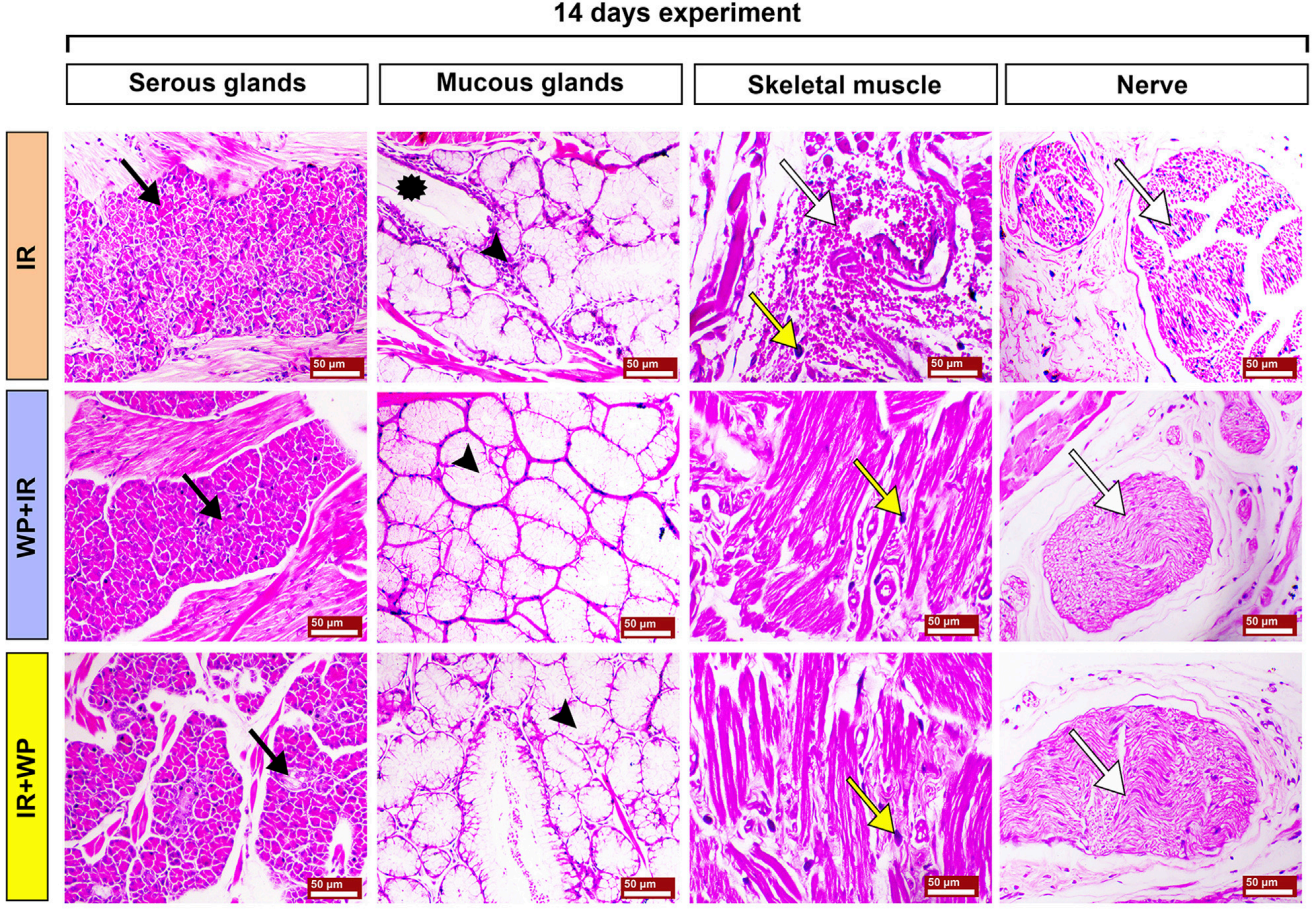
Photomicrographs of the subepithelial structures of the tongues of different groups of rats sacrificed at 14 days post-irradiation. The IR group shows severe apoptosis of the serous glands (black arrow), marked mucous retention (asterisk) associated with mononuclear inflammatory cell infiltration (arrowhead); the muscle fibers displayed interstitial hemorrhage (white arrow), mast cell infiltration (yellow arrow), and splitting of the nerve fibers (white arrow). The WP+IR group shows normal serous glands (black arrow), mucous glands (arrowhead), mild myolysis with mild infiltration of mast cells (yellow arrow), and normal nerve bundles (white arrow). The IR+WP group exhibits focal degenerative changes in serous glands (black arrow), moderate retention of the mucous (arrowhead), focal myolysis, a decrease in the infiltration of mast cells (yellow arrow), and normal nerve bundles (white arrow). H&E stain, bars = 50 µm.

The histoarchitecture modification in the lingual tissues following IR exposure and/or WP administration was examined in order to emphasize the aforementioned findings (Figure 6; Figure 7; Figure 8; Figure 9). As depicted in Figure 6, the tongue histological examination of the control and WP groups exhibited normal architectures of all tongue papillae. The filiform papillae are cone-shaped and covered by the well-keratinized stratified squamous epithelium with evident keratohyalin granules in the granular cell layer. The fungiform papillae are normally mushroom-shaped with intra-epithelial taste buds on the top. The circumvallate papillae are barrel-shaped and contain taste buds on both sides of the trough. In contrast, the IR-exposed rats exhibited distortion in the histologic appearance of tongue tissues. Filiform papillae were distorted with marked desquamation. The taste bud cells of fungiform papillae and circumvallate papillae were distorted and disorganized, losing their boundaries, and showed nuclear pyknosis. The WP + IR group showed a reduction in side effects induced by IR exposure, evidenced by decreased degenerative and necrotic lesions within the filiform papillae with the apparent continuous eosinophilic keratin layer. Fungiform and circumvallate papillae exhibited less degenerative changes within the taste buds on the top surface and on both sides of the trough, respectively. Rats treated with WP exhibited lesser amendment of IR damage than the protected group. The filiform papillae lost their typical conical shape; however, the taste buds of the fungiform and the circumvallate papillae showed little degenerative changes.

Histological alterations of tongue papillae of rats sacrificed 14 days post-RT are depicted in Figure 7. The IR-exposed animals demonstrated exaggerated effects of irradiation after a longer duration. Filiform papillae appeared necrotic and completely sloughed with keratinolysis. Fungiform papillae showed marked degeneration of their taste buds with hyperkeratosis of the neighboring epithelium. The cells of taste buds in the trough of the circumvallate papillae were degenerated and atrophied. Wide edematous spaces were clearly seen in lamina propria. Mononuclear inflammatory cells, comprised of lymphocytes and macrophages, were observed. WP + IR showed a reduction in tongue tissue distortion to a lesser extent than in rats subjected to IR only. Animals of this group showed a significant decrease in oral epithelial tissue injury associated with mild loss of their filiform papillae and with apparent intact taste cells of the taste buds within both fungiform and circumvallate papillae. Rats in the IR + WP group exhibited focal sloughing of the filiform papillae and less atrophied taste buds.

The sections of the lingual submucosa of the different groups sacrificed on day 7 are illustrated in Figure 8. The control and WP groups showed a normal architecture. The glands appeared to be composed of normally arranged serous and mucous cells. The serous secretory cells were stained deeply with basophilic dyes, while the mucous cells exhibited pale staining with the bubbly cytoplasm and basal flat nuclei. The minor glands displayed interlobular ducts with some serous demilunes that capped mucous acini. The intrinsic striated muscle fibers showed bundles of muscle fibers with peripheral nuclei. Nerve bundles were found to be composed of nerve fibers with zigzag morphology due to shrinkage during histologic section preparation. The bundles were surrounded by epineurium and separated by connective tissue. In contrast, IR-subjected rats exhibited degeneration and atrophy of the serous lobules and acini. The excretory ducts were partially occluded with stagnant secretory material. Mucous acini exhibited less evident changes with degeneration of serous demilunes. The striated ducts of minor mucous glands were significantly dilated with atrophy of the epithelial lining. The striated muscle fibers revealed sarcoplasmic degenerative changes associated with fiber atrophy, marked interstitial edema, and mast cell infiltration. Nerve bundles displayed atrophy, fragmentation, vacuolation of nerve fibers with hypertrophied Schwann cells, and perineural infiltration of mast cells. However, rats in the WP + IR group depicted a marked decrease in degenerative changes of the serous and mucous glands. The muscle fibers showed focal sarcoplasmic degeneration and decreased edema and mast cell infiltration, alongside mild neuronal vacuolation. Animals treated with WP after IR revealed focal necrosis of few serous glands, decreased stagnant mucous within the mucous acini, moderate degeneration and fragmentation of the muscle fibers, and slight signs of vacuolation and degeneration of the nerve bundles with perineural edema.

In addition, Figure 9 illustrates the histological changes in lingual submucosa of rats sacrificed on day 14 after IR exposure. The IR group showed severe degeneration and apoptosis of the serous glands. The mucous glands exhibited degenerative changes associated with mucin retention and mononuclear inflammatory cell infiltration. Extensive atrophy of muscle fibers with marked interstitial hemorrhage and mast cell infiltration was observed. In addition, splitting of the nerve fibers was also noted. Conversely, the WP + IR group demonstrated a decrease in the degenerative changes within the glandular, muscular, and neural tissues, with only mild myolysis and mild infiltration of mast cells within the striated muscle fibers. On the other hand, the IR + WP group showed focal degenerative changes in serous and mucous glands with a decrease in the stagnant secretion within their acini, decreased atrophy of muscle fibers, and decreased infiltration of mast cells. Nerve bundles appeared unaltered. Regarding the overall lesion score in the context of Table 2, the IR group showed a time-dependent increase in the oral lesions, the WP + IR group showed alleviation of the oral lesions compared to the IR group after sacrifice at day 7, and both WP + IR and IR + WP groups showed a significant decrease in the lesion score compared to the IR group.

**TABLE 2:** Histopathological score upon IR exposure and/or WP supplementation in lingual tissues.

Day	Control	WP	IR	WP + IR	IR + WP
Day 7	ND	ND	2.50 ± 0.29	0.75 ± 0.25***	1.50 ± 0.29*
Day 14	ND	ND	3.75 ± 0.25	1.00 ± 0.00**	1.75 ± 0.25*

Data presented as means ± SE.

ND, not detectable lesions; *p < 0.05, **p < 0.01, and ***p < 0.01 vs. IR group.

### 3.5 Biological networks, hierarchical heatmap, and VIP score

**FIGURE 10:**
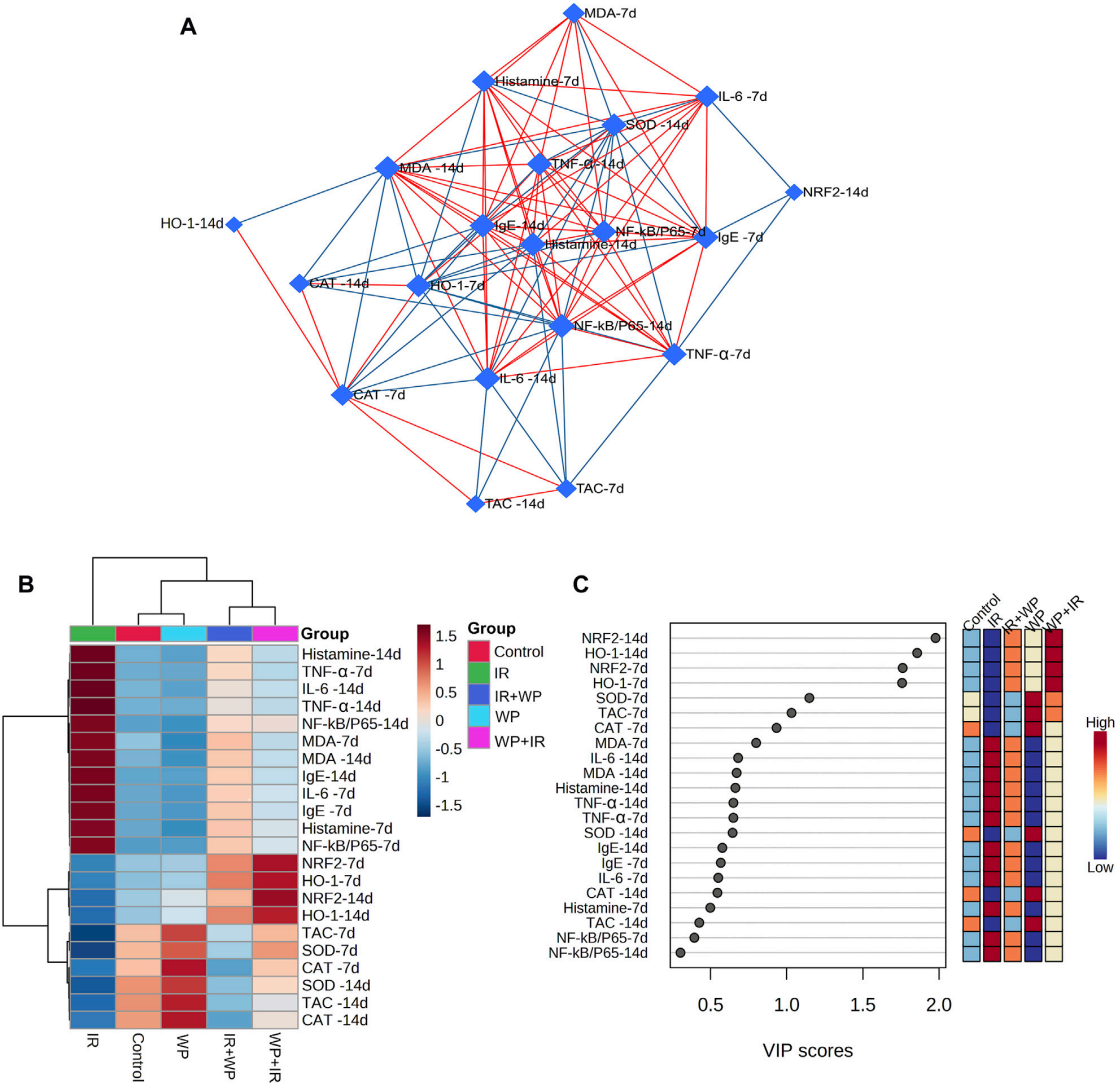
Clustering analysis of whole datasets after IR and/or WP exposure. **(A)** DSPC network of significantly different variables in the IR-exposed and control groups. The measured variables are represented by the nodes in the DSPC network, while the correlation measures are represented by the edges. Stronger correlated variables tend to cluster together and have larger borders between them. The blue lines display a negative correlation, whereas the red lines display a positive correlation with variables. **(B)** Heatmap and hierarchical clustering provide a visual summary of all the data. The rows and columns of the map are made up of various averages and treatment sets, respectively, and each colored cell on the map indicates a concentration value. On the gradation scale, dark red has the highest value, while blue has the lowest. **(C)** VIP score, the average concentrations of the measured variables are displayed for each study group in colored boxes on the right, and a colored scale from maximum (red) to least (blue) represents the contribution strength.

Multivariate analyses were conducted to ascertain the association amongst various variables and treatments, as shown in Figure 10. Biological networks of all variables were created. The nodes depict different parameters, while the lines display the relationships between these various variables in the debiased sparse partial correlation (DSPC) algorithm network (Figure 10A). During the data normalization stage, the data were transformed into log or cubic roots for optimal performance. The variable that is closer to the center reveals a stronger correlation with these selected variables and the more relevant position in the network, such as histamine on day 14. On the other hand, histamine is positively correlated with the majority of examined parameters on day 14 while negatively correlated with HO-1, CAT, SOD, and TAC on day 7. The metabolic pathway network was also constructed to explore the relationships between the most disrupted pathways and various parameters induced by IR exposure.

The clustering heatmap explicates a clear visual depiction of all datasets (Figure 10B) and reveals a notable discrepancy in concentration levels of whole measured variables in response to IR exposure compared to other groups.

Furthermore, according to the VIP score, the top influential factors in our study were NRF2 and HO-1 (days 7 and 14), followed by SOD, TCA, and CAT (day 7), which were sensitive to different treatments and could distinguish the IR-exposed group from others (Figure 10C).

## 4 Discussion

When IR is used as a main or adjuvant therapy for head and neck cancer, it inevitably exposes nearby healthy soft tissues to undesirable injury (Pandya et al., 2014). The tissues with high cellular turnover tend to be the most radiosensitive, especially the tongue, which displays severe mucous membrane damage (Üstün et al., 2014). Many compelling publications have revealed that radiation-caused damage is principally a consequence of complex interactions among a variety of processes including the overwhelming production of ROS and inflammatory mediators (Ugurluer et al., 2016; De Ruysscher et al., 2019; Allegra et al., 2020). Overproduction of ROS causes an imbalance between the cellular pro-oxidants (O_2_^•−^, H_2_O_2_, OH^•^, and NO) and antioxidant enzymes, thus altering nucleic acid integrity, membrane cohesion, cellular protein, and, ultimately, causing cell death (Allegra et al., 2020; Abdeen et al., 2021; Elgazzar et al., 2022).

Herein, our present study elucidated that oxidative stress was crucial in the lingual damage caused by IR, indicated by the decline of the SOD and CAT activities, alongside the TAC level, in the tongue of exposed rats. SOD is an endogenous enzyme serving as the first line of defense that swiftly catalyzes the dismutation of O_2_^•−^ to O_2_ and H_2_O_2_. Thereafter, CAT degrades the generated H_2_O_2_ into H_2_O and O_2_. Nevertheless, when CAT is dwindling, an iron-catalyzed Haber–Weiss reaction (H_2_O_2_+ Fe^2+^ → OH^•^ + OH^−^ + Fe^3+^) generates extremely harmful reactive OH^•^ radicals that can directly damage the membrane lipids, triggering LPO with a further generation of another harmful substance, MDA (an LPO biomarker) (Giuranno et al., 2019; Abdelnaby et al., 2022). Even worse, MDA itself could interact with other key biomacromolecules, resulting in extensive cellular damage (Abdelnaby et al., 2022). Current investigation revealed substantial increases in MDA levels, confirming the possibility of membrane insult following IR exposure. Our oxidative stress parameters were comparable with prior investigations that showed that cellular antioxidants were decreased upon exposure to irradiation (El-Desouky et al., 2017). Additionally, the aforementioned findings coincide with those of Ugurluer et al. (2016) who confirmed boosted MDA levels upon IR exposure. Increased LPO corresponded with current lingual histology showing the degenerative changes within the lingual papillae and submucosal tissues.

Further evidence of the promotion of oxidative damage upon IR exposure in our investigation was the downregulation of NRF2 and HO-1 mRNA transcripts in the tongue tissue. The NRF2 signaling pathway is a well-known key regulator of the cellular detoxification mechanism and the redox condition. It boosts the capacity of cellular antioxidants such as HO-1, CAT, and SOD (Habotta et al., 2023). Our histological findings of lingual sections strikingly mirrored these findings, where they demonstrated exaggerated distortion in the histologic appearance of tongue tissues after days 7 and 14 post-irradiation in a time-dependent pattern. All tongue tissues, including papillae, glands, muscles, and nerves, showed signs of degeneration and degeneration and atrophy. In the same vein, Pandya et al. (2014) observed a significantly higher number of apoptotic bodies in irradiated tissue than the control cases.

Radiation-provoked oral mucositis is the major drawback of IR of oral malignancy that has an impact on a cancer survivor’s quality of life (Pandya et al., 2014; Subramaniam and Muthukrishnan, 2019). Since there is ample evidence that oxidative stress and inflammation are closely related, we confirm that the inflammatory response is another mechanism triggering harm from IR. ROS and DNA damage will initiate an intracellular signaling cascade and boost the expression of pro-inflammatory genes, leading to the release of inflammatory mediators, culminating in a profound inflammatory response (Shanab et al., 2023). According to the outcomes of the current investigation, IR significantly increases serum levels of IgE, histamine, and pro-inflammatory cytokines (IL-6 and TNF-α). Furthermore, NF-κB/p65 m-RNA gene expression was upregulated. Following exposure to IR, IgE-mediated mast cell activation triggers the production of histamine and causes increased expression of histamine and inflammatory cytokines (Landy et al., 2019; Tanaka and Furuta, 2021). Histamine serves a crucial role in the local tissue inflammatory reaction to IR by increasing NF-κB/p65 activation and inflammatory cytokine production (Landy et al., 2019). The NF-κB/p65 pathway is a redox-regulated transcription factor and is essential for the development of mucositis (Zhang et al., 2021). TNF-α is the most significant inflammatory mediator that triggers the activation of IL-6 and adhesion molecules, which promote the migration of leukocytes into the inflammatory zone (Ahmed et al., 2022). Congruent with this assertion is a study by Citrin and Mitchell (2017) that asserted IR can rapidly escalate inflammatory responses via a plethora of cytokines in irradiated tissues. Current histological findings of lingual tissue vividly corroborated these effects, as elucidated by desquamative lesions of tongue papillae with substantial mononuclear inflammatory cells and mast cell spillage in IR-injured tongue tissues. Similar results have been reported by Zhang et al. (2021) who found basement membrane disruption, epithelial loss, and ulceration in irradiated tissue.

Xerostomia is the most prominent complication in patients receiving head and neck radiotherapy. Destruction of the salivary glands and their associated nerves is one of the contributing factors to IR-triggered xerostomia. Salivary glands are incredibly vulnerable to radiation; IR causes alteration in the volume of saliva, its consistency, and its pH (Basu et al., 2012). These findings are reflected in the current histological investigation, where the lobules of the salivary glands displayed atrophic changes and their ducts and acini were partially blocked by stagnant secretory material, suggesting a potential decline in salivary flow, promoting xerostomia.

Moreover, most head and neck cancer patients receiving IR suffer from impaired taste perception and frequently complain of being incapable of tasting their food, decreasing food intake (Deshpande et al., 2018). These findings were confirmed in our histology as we observed massive atrophy of taste bud cells and nerves. These results agreed with Nguyen et al. (2012) who reported that, within 1–3 days of irradiation, taste progenitor cells experience cell cycle arrest or apoptosis. This could be explained by the extreme radiosensitivity of the taste buds and taste nerves (El-Rouby and El-Batouti, 2021).

WP has been proven to have therapeutic potential, besides being nutritive. It is frequently used in conventional medicine, which is primarily credited to the biological functions of its bioactive peptides (Zou et al., 2016; Pezeshki et al., 2023). The peptides surpass the functional attributes of their parent proteins and have been paid great attention due to health-promoting benefits, such as antioxidant, anti-inflammatory, immunomodulatory, antibacterial, and anticancer activities (Liu et al., 2021). As a result, protein-derived bioactive molecules not only help cancer patients satisfy their protein needs but also produce more biological activities that can help repair damage caused by IR (Liu et al., 2021). Accordingly, FTIR spectroscopy of the WP was done in our study to estimate protein secondary structures. The results revealed that α-helix and β-sheet were the most prevalent secondary structural components of protein. Our results concur with the information provided by Wang et al. (2019). Evidence indicates that α-helices and β-sheet secondary structures are involved in the structure of antioxidant peptides which are crucial for their antioxidant properties (Zou et al., 2016).

In the current study, WP consumption weakened IR-induced lingual damage as shown by the noteworthy increase in the antioxidant enzyme activity through upmodulating the expression of oxidative stress genes (NRF2 and HO-1), along with mitigation of LPO. Furthermore, our histopathological examination revealed that WP had a more powerful protective action against IR-induced damage in the irradiated tongue than using it as a treatment modality after irradiation. The antioxidant capability of WP is pertaining to its abundance of cysteine (a thiol group-containing amino acid) that combines with glutamate and other amino acids to form GSH. Subsequently, TAC contents are increased, boosting the scavenging of free radicals generated by IR (El-Desouky et al., 2017; Falkowski et al., 2018). In addition, the hydrolysis of WP results in the generation of bioactive peptides that have antioxidant properties. Furthermore, the scavenging capacity of tyrosine and cysteine amino acid residues in WP is primarily reliant on proton-coupled single electron or hydrogen atom transfer processes (El-Desouky et al., 2017). In accordance with our data, Giblin et al. (2019) and Veskoukis et al. (2020) reported that WP boosts the antioxidant profile and improves the redox state.

In addition, in the current study, WP exhibited potent anti-inflammatory effects via restoration of serum levels of histamine, TNF-α, and IL-6, along with downregulation of NF-κB/p65 expression in tongue tissue. The most active fractions of WP extracts are β-lactoglobulin and α-lactalbumin, which collaborated to increase neutrophils’ sensitivity to a subsequent stimulus (Rusu et al., 2009). WP exhibits anti-inflammatory activities by reducing the expression of the TNF-α gene (Pal and Ellis, 2010). WP has been shown to decrease the inflammatory reaction by constraining the NF-κB/p65 trajectory (Ali et al., 2022). In the same vein, Liu et al. (2021) reported that WP effectively decreased the pro-inflammatory cytokine levels and alleviated the inflammatory reaction. The present histopathological findings revealed a decrease in the desquamative lesions and in mast inflammatory cell infiltration in lingual tissues when WP was used in IR-exposed animals, with better amendment of irradiation damage if used before exposure, in a time-dependent pattern.

**FIGURE 11:**
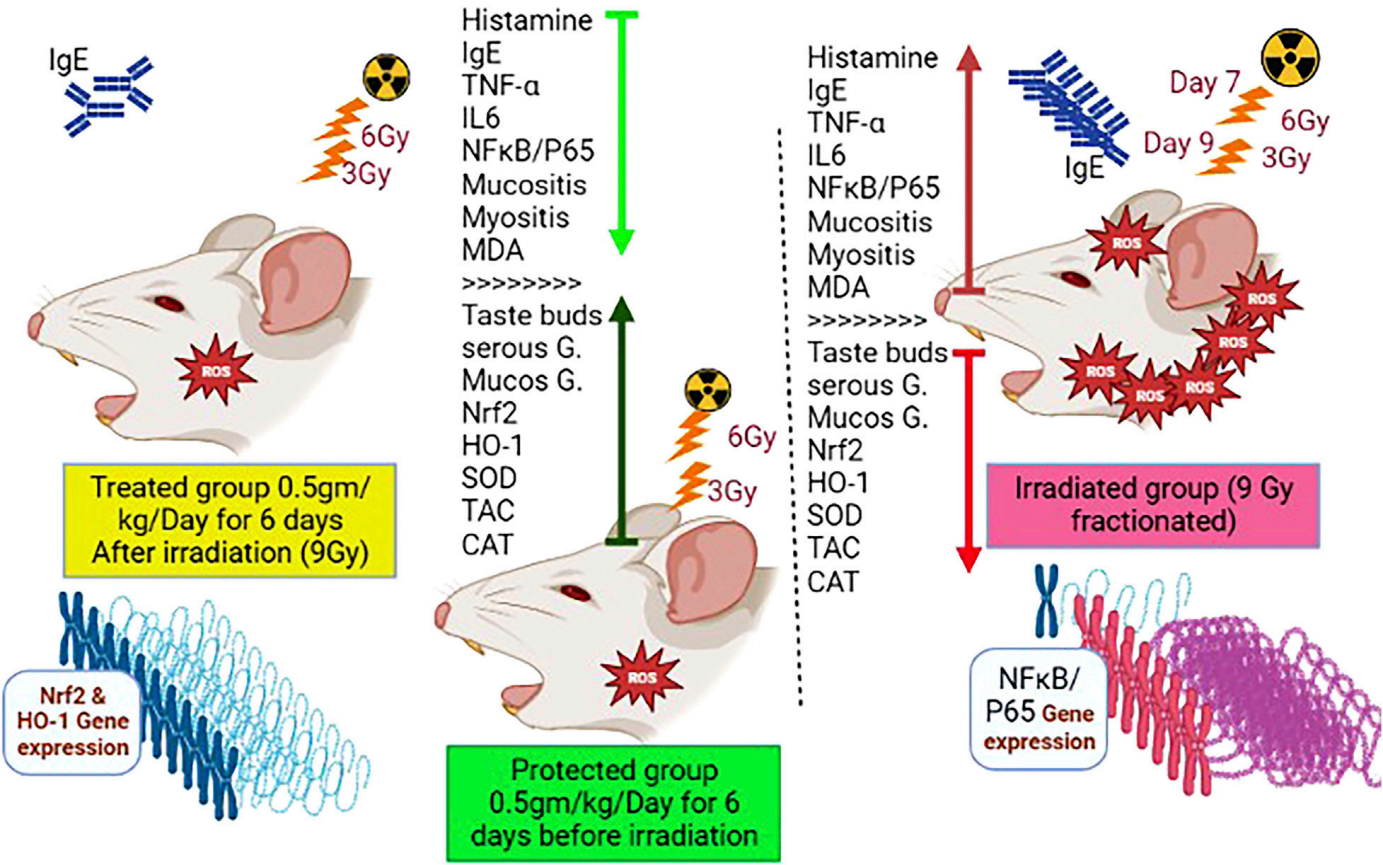
Molecular mechanisms underpinning WP capacity to protect against tongue injury caused by IR exposure.

In order to gather the variable contributions influenced by various treatments on rat lingual tissue, multivariate statistical analysis using the DSPC network, clustering heatmap, and VIP score was also performed. In addition, histamine on day 14 was shown to have a more relevant position in the DSPC network and to be positively associated with most of the parameters that were looked at while being negatively correlated with HO-1, CAT, SOD, and TAC on day 7. The clustering heatmap clearly illustrates that IR exposure caused substantial changes in all tested parameters compared to other treatment groups, pointing to potential adjustments in such parameters following WP supplementation. NRF2 and HO-1 (days 7 and 14) were also found to be the top impacting variables in our study according to the VIP score. The molecular mechanisms underpinning WP capacity to protect against tongue injury caused by IR exposure are depicted in Figure 11.

## 5 Conclusion

Gamma irradiation provoked notable harmful effects on the lingual structures of rats caused by oxidative stress and inflammatory reaction in a time-dependent pattern. WP supplementation could abrogate IR-induced lingual tissue damage. This betterment has been proposed for WP antioxidant and anti-inflammatory properties. When WP was given before irradiation, it resulted in a higher improvement of IR-induced damage than WP given post-exposure. Our findings advocate the addition of WP to foods of IR-treated cancer patients, owing to its protective and remedial effects toward IR-triggered tongue damage. However, further investigations of the current hypothesis are encouraged, particularly in clinical cases.

## Data Availability

The original contributions presented in the study are included in the article/Supplementary Material; further inquiries can be directed to the corresponding authors.
